# 

**Published:** 1891-02

**Authors:** 


### CONTENTS:

ARTICLE I.—The Treatment of Proximate Cavities. By George Howe Winkler, M.D., D.D.S. - - - - 433
" II.—Treatment of the Third Stage of Alveolar Abscess. By E. C. Moore, D.D.S. - - - - - 437
" III.—The Treatment of Proximate Surfaces. By George A. Wilson, D.D.S. - - - - - - 440
" IV.—Olden-Time Artificial Teeth. - - - - - 447
" V.—The Application of Massage in the Treatment of Dental Pathological Conditions. By. W. F. Rehfuss, D.D.S. - - - - - - 450
" VI.—Some Practical Points Involved in the Relation of the Upper to the Lower Teeth. - - - - 455
" VII.—The Dangers arising from Syphilis in the practice of Dentistry. By L. Duncan Bulkley, A,M.,M.D. 461
" VIII.—Simple Method for Controlling Epistaxis. - - 465
" IX.—The Combination of Tin and Gold as a Filling Material. By Dr. H. F. Hamilton. - - - 467

### EDITORIAL.

A Tooth Drawn by a Hen. - - - - - - 469
Antiquity of Aritficial Teeth. - - - - - - 469
A Remarkable Surgical Operation. - - - - - 470
The Youngest Doctor. - - - - - - - - 470
The Malleabilitv of Gold. - - - - - - 470
An Electric Plant. - - - - - - - - 471

### MONTHLY SUMMARY.

Practical Suggestions. - - - - - - - - 471
The Nature of Koch's Lymph. - - - - - 472
Coffee as a Disinfectant. - - - - - - - - 473
Chloroform as an Antiseptic. - - - - - - 474
Physiological Action of Thialdine. - - - - - 475
A Ready Inhaler. - - - - - - - 476
Cures for Neuralgia of the Head. - - - - - 476
Campho-phenique. - - - - - - - - 477
Administration of Morphine by the Nostrils. - - - - 478
Codein. - - - - - - - - - - - 479
A New Mixture for Use in Local Anæsthesia. - - - - 479
Isococaine, a New Anæsthetic. - - - - - - 480

